# The high-cost, type 2 diabetes mellitus patient: an analysis of managed care administrative data

**DOI:** 10.1186/2049-3258-72-6

**Published:** 2014-02-27

**Authors:** Juliana L Meyers, Shreekant Parasuraman, Kelly F Bell, John P Graham, Sean D Candrilli

**Affiliations:** 1 RTI Health Solutions, 200 Park Offices Drive, Research Triangle Park, NC 27709, USA; 2 AstraZeneca, 1800 Concord Pike, Wilmington, DE 19850, USA; 3 Bristol-Myers Squibb, 777 Scudders Mill Road, Plainsboro, NJ 08536, USA

**Keywords:** Diabetes mellitus, type 2, Health care costs, Economics

## Abstract

**Background:**

Type 2 diabetes mellitus (T2DM) affects 25.8 million individuals in the United States and exerts a substantial economic burden on patients, health care systems, and society. Few studies have categorized costs and resource use at the patient level. The goals of this study were to assess predictors of being a high-cost (HC) patient and compare HC T2DM patients with not high-cost (NHC) T2DM patients.

**Methods:**

Using managed care administrative claims data, patients with two or more T2DM diagnoses between 2005 and 2010 were selected. Patients were followed for 1 year after their first observed T2DM diagnosis; patients not continuously enrolled during this period were excluded from the study. Study measures included annual health care expenditures by component (i.e., inpatient, outpatient, pharmacy, total). Patients accruing total costs in the top 10% of the overall cost distribution (i.e., patients with costs > $20,528) were classified as HC a priori; all other patients were considered NHC. To assess predictors of being HC, a logistic regression model was estimated, accounting for demographics; underlying comorbidity burden (using the Charlson Comorbidity Index [CCI] score); diagnoses of renal impairment, obesity, or hypertension; and receipt of insulin, oral antidiabetics only, or no antidiabetics.

**Results:**

A total of 1,720,041 patients met the inclusion criteria; 172,004 were HC. The mean (SD) CCI score for HC patients was 4.3 (3.0) versus 2.1 (1.7) for NHC patients. Mean (SD; upper 95% confidence interval-lower 95% confidence interval) annual per-patient costs were $56,468 ($65,604; $56,778-$56,157) among HC patients and $4,674 ($4,504; $4,695-$4,652) among NHC patients. Inpatient care and pharmacy costs were higher for HC patients than for NHC patients. The strongest predictor of being an HC patient was having a CCI score of 2 or greater (odds ratio [OR] = 4.896), followed by a diagnosis of obesity (OR = 2.106), renal impairment (OR = 2.368), and insulin use (OR = 2.098).

**Conclusions:**

High-cost T2DM patients accrue approximately $52,000 more in total annual health care costs than not high-cost T2DM patients. Patients were significantly more likely to be high-cost if they had comorbid conditions, a diagnosis of obesity, or used insulin.

## Background

Diabetes is a chronic, progressive condition that, if not managed properly, can lead to numerous health complications and disability. It has been estimated that approximately 25.8 million people in the United States (US) have diabetes [1]. In adults, 90% to 95% of patients with diabetes have type 2 diabetes mellitus (T2DM), which is characterized by insulin resistance, pancreatic beta-cell dysfunction, and excessive glucose production by the liver. Over the next 40 years, the total prevalence of diabetes in the US is expected to more than double, from 5.6% in 2005 to 12.0% in 2050 [2].

Treatment of T2DM consists of significant lifestyle adjustments and drug therapy, including oral antidiabetic agents and insulin therapy [3]. The American Diabetes Association and the European Association for the Study of Diabetes have agreed that early intervention with metformin should be used in patients with hyperglycemia to help maintain recommended levels of glycemic control [4]. Because T2DM is a progressive disease, timely augmentation of therapy with additional agents, such as insulin, sulfonylureas, thiazolidinediones, dipeptidyl peptidase-4 inhibitors, and glucagon like peptide-1 receptor agonists, also is recommended [5].

It is well established that the costs attributable to diabetes (including T2DM) are substantial. One recent estimate suggested that the total economic burden of all types of diabetes in the US exceeds $174 billion annually, which includes $116 billion in excess medical expenditures and $58 billion in reduced nation productivity [6]. Medical costs attributed to all types of diabetes include $27 billion for direct care, $58 billion to treat patients with diabetes-related chronic complications, and $31 billion in excess general medical costs [6].

Significant evidence exists showing the relationship between diabetes-related costs and observed glucose values [7-12]. However, as informative as these studies have been in communicating the economic impact of diabetes, many studies have included data from sources outside the US or did not focus on managed care populations. Further, identifying T2DM patients who could be considered high cost (HC) is of significant interest to health care payers, given the rising health care costs in the US—interventions could be developed that would focus on patients who are likely to become HC and therefore minimize costs of the disease. Previous studies have identified HC patients in other disease areas (e.g., acute coronary syndromes) [13]; but to our knowledge, no retrospective analysis has been published that examines HC patients with T2DM.

The goal of this analysis was to document the actual health care costs incurred by payers for a T2DM population and to determine which factors were associated with patients in the higher tiers of the T2DM cost distribution. Additionally, this study compared health care costs for patients who were identified as HC with patients who were identified as not high cost (NHC).

## Methods

This analysis used the LifeLink database (formerly PharMetrics), a commercially available source of computerized administrative claims from 95 managed care health plans covering more than 61 million unique patients. The database included claims from private health plans in all four US geographic regions and had an age and sex distribution representative of national managed care enrollment. The database included patient-level demographics and periods of health plan enrollment; primary and nonprimary diagnoses; detailed information about hospitalizations, diagnostic testing, and therapeutic procedures; inpatient and outpatient physician services; prescription drug use; and cost data in the form of managed-care reimbursement rates for each service. Data are tracked longitudinally within patients via de-identified and unique patient numbers. For the purposes of this analysis, the most recent 5 years of data were used. RTI International’s institutional review board determined that this study met all criteria for exemption. Data were available from January 1, 2005, through December 31, 2010.

All patients with at least two diagnoses of T2DM (*International Classification of Diseases, Ninth Revision, Clinical Modification* [ICD-9-CM] codes 250.×0 and 250.×2) during the selection window (January 1, 2005, through December 31, 2009) were initially identified for study inclusion. Requiring two T2DM diagnoses helped to rule out patients who may have been suspected of having T2DM but were never formally diagnosed and likely eliminated patients who were miscoded as having T2DM. For each patient identified, his or her index date was defined as the date of the first observed T2DM diagnosis. To allow for adequate follow-up time to address the research questions and to ensure that any observed lack of health care events was the result of no medical activity and not the result of cessation of care, patients were required to have at least 12 months of continuous post-index date observation. Furthermore, to obtain the largest possible sample size of patients with T2DM, we did not require patients to have received a diabetic medication, nor was any pre-index date enrollment required.

Subgroups of patients in the T2DM population were identified as HC or NHC on the basis of where their annual costs fell within the overall T2DM cost distribution. We identified two subgroups of HC T2DM patients a priori: those patients whose costs were in the 90th or greater percentile of all-cause costs and those patients whose costs were in the 80th or greater percentile of all-cause costs. We also identified two subgroups of NHC T2DM patients: those patients whose costs were below the 90th percentile of all-cause costs and those patients whose costs were below the 80th percentile of all-cause costs. We chose to assess both the top 10% and top 20% of patients in the all-cause cost distribution for several reasons. First, it is a well-regarded rule of thumb that the top 20% of patients accrue 80% of health care costs [14]. We wished to evaluate whether this rule held true in a T2DM population. Second, previous studies examining HC patients in other disease areas have used similar methodology [13]. We also felt that it was important to see if patient characteristics changed as we went from the top 20% of patients to the top 10% of patients. For this analysis examining HC and NHC T2DM patients, all-cause costs were examined (rather than T2DM-related costs) because many patients likely had coexisting conditions (e.g., hypertension or renal impairment) that were T2DM related but that may not have been coded as such in the claims.

HC patients then were compared with NHC patients. Key outcome measures that were analyzed included patient characteristics, overall (all-cause) health care resource use and costs, and T2DM-related resource use and costs. All outcome measures presented in this paper were assessed over a 1-year post-index date follow-up period, unless stated otherwise. Patient characteristics that were assessed at the index date included age, sex, geographic region, insurance payer type (i.e., commercial, Medicare, Medicaid, self, Medicare Gap, and missing/unknown), and health plan type (i.e., health maintenance organization, preferred provider organization, point of service, indemnity, and missing/unknown). To assess overall comorbidity burden, we calculated a Charlson Comorbidity Index (CCI) score for each patient over the 1-year follow-up period. The mean CCI score, along with the number and percentage of patients with a score < 2 or ≥ 2, was reported. The CCI score included 17 categories of comorbidities, as defined by ICD-9-CM diagnosis and procedure codes, with associated weights corresponding to the severity of the comorbid condition of interest [15]. Because all patients in the study had a diagnosis of T2DM and because we wished to evaluate underlying comorbidity burden independent of T2DM, comorbidities corresponding to T2DM were removed from the CCI score (i.e., we did not want the CCI to be inflated for all patients). Antidiabetic agents received were reported by class (i.e., sulfonylureas, meglitinide, biguanide, thiazolidinedione, alpha-glucosidase inhibitor, dipeptidyl peptidase-4, glucagon-like peptide-1, and insulin).

For each patient, overall health care utilization and associated costs were aggregated across all encounters, regardless of reason, observed during the 1-year post-index date period. The following categories of overall health care utilization and costs were evaluated and reported: inpatient, skilled nursing facility, emergency department, outpatient hospital, office visit, laboratory service, other outpatient care, pharmacy, and total health care utilization. For each category of overall health care utilization, the number and percentage of patients with a visit or admission, the mean (standard deviation [SD]) number of visits or admissions, and the per-patient costs were reported. Additionally, for patients with an inpatient or skilled nursing facility admission, the average number of days per admission was reported.

The total volume and associated costs of health care services specifically related to T2DM also was reported. Hospital admissions related to T2DM were identified by searching for inpatient hospital confinements in which T2DM was recorded as the primary discharge diagnosis (i.e., ICD-9-CM codes 250.×0 and 250.×2). Office, emergency department, outpatient hospital, other outpatient visits, and laboratory services related to T2DM were identified by searching for medical claims with any diagnosis (i.e., primary or secondary) of T2DM and a relevant place-of-service code for the care setting of interest. Additionally, we evaluated the use and associated costs of all disease-specific medications for which a claim was generated. Medications were identified using appropriate National Drug Codes and Healthcare Common Procedure Coding System codes, brand and generic product names, and therapeutic class descriptions provided in the database.

All analyses were carried out using SAS (Version 9; Cary, North Carolina) statistical software. Descriptive analyses entailed the tabular display of mean and SDs for continuous variables of interest (e.g., total health care costs) and the frequency distribution of categorical variables of interest (e.g., health plan type). All-cause and diabetes-related health care costs were updated to 2011 US dollars, using the medical care component of the US Consumer Price Index.

Logistic regression models were estimated to assess predictors of being an HC patient with T2DM (separate models for the top-10% and the top-20% groups). The dependent variable was a dichotomous (i.e., 0 or 1) variable indicating whether the patient was in the HC cohort. Demographic characteristics that have repeatedly been shown to be associated with costs were used as independent variables and included patient age (i.e., < 35 years, 35–44 years, and 45–54 years vs. ≥ 55 years), sex (i.e., male vs. female), geographic region (i.e., South, Midwest, and West vs. East), health plan type (i.e., preferred provider organization, point of service, indemnity, and missing/unknown vs. health maintenance organization), and payer type (i.e., Medicaid, Medicare, self, Medicare Gap, and missing/unknown vs. commercial). Clinical variables available in the database were also selected as independent variables and included the CCI score (i.e., CCI score < 2 vs. CCI score ≥ 2), the types of pharmacologic treatments the patient received (i.e., insulin and oral antidiabetic medications vs. no pharmacological treatment), a diagnosis of renal impairment (i.e., had a renal impairment diagnosis vs. did not have a renal impairment diagnosis), and a diagnosis of obesity (i.e., had an obesity diagnosis vs. did not have an obesity diagnosis). Patients with missing age, sex, health plan, and health payer information were excluded from the regression models.

## Results

Among the 1.72 million T2DM patients in the database who met the initial study inclusion and exclusion criteria, 344,019 were identified as being in the top 20% of the cost distribution (i.e., costs > $10,901), and 172,004 were identified as being in the top 10% of the cost distribution (i.e., costs > $20,528) (Table 1). Mean (SD) patient age among patients in the top 20% of the cost distribution was 57.2 (13.7) years versus 57.7 (14.9) years among patients in the bottom 80% of the cost distribution. In both the top 20% and the bottom 80% of patients, sex distribution was approximately equal. The mean (SD) CCI score was greater among patients in the top 20% of the cost distribution (3.7 [2.8]), than among patients in the bottom 80% of the cost distribution (2.0 [1.6]). Chronic pulmonary disease, liver disease, and congestive heart failure were the most common conditions in both cohorts. The percentage of patients receiving oral antidiabetic medications at index date was approximately the same in both cohorts; however, more than twice the percentage of patients in the top 20% of the cost distribution received insulin, compared with patients in the bottom 80% of the cost distribution (8.7% vs. 4.0%).

**Table 1:** Characteristics of the study sample, by cohort

	**Cohort**							
	**Top 10% of costs**		**Bottom 90% of costs**		**Top 20% of costs**		**Bottom 80% of costs**	
	**N**	**%**	**N**	**%**	**N**	**%**	**N**	**%**
Total sample	172,004		1,548,037		344,019		1,376,022	
Age (years)								
< 18	1,194	0.69%	17,232	1.11%	3,469	1.01%	14,957	1.09%
18-25	1,445	0.84%	17,163	1.11%	3,269	0.95%	15,339	1.11%
25-34	4,920	2.86%	56,918	3.68%	11,866	3.45%	49,972	3.63%
35-44	14,922	8.68%	164,584	10.63%	31,878	9.27%	147,628	10.73%
45-54	40,506	23.55%	359,704	23.24%	80,397	23.37%	319,813	23.24%
55-64	69,775	40.57%	474,253	30.64%	130,935	38.06%	413,093	30.02%
≥ 65	39,168	22.77%	457,026	29.52%	82,028	23.84%	414,166	30.10%
Missing/unknown	74	0.04%	1,157	0.07%	177	0.05%	1,054	0.08%
Mean (SD)	57.46	(12.89)	57.61	(14.82)	57.17	(13.66)	57.7	(14.87)
Sex								
Male	86,359	50.21%	789,524	51%	164,587	47.84%	711,296	51.69%
Female	85,630	49.78%	758,425	48.99%	179,402	52.15%	664,653	48.30%
Missing/unknown	15	0.01%	88	0.01%	30	0.01%	73	0.01%
Geographic region								
East	55,618	32.34%	521,500	33.69%	113,485	32.99%	463,633	33.69%
South	36,130	21.01%	371,487	24%	71,733	20.85%	335,884	24.41%
Midwest	55,462	32.24%	444,249	28.70%	110,809	32.21%	388,902	28.26%
West	24,794	14.41%	210,801	13.62%	47,992	13.95%	187,603	13.63%
Health plan type								
Health maintenance organization	37,332	21.70%	293,868	18.98%	73,580	21.39%	257,620	18.72%
Preferred provider organization	96,019	55.82%	873,018	56.40%	190,440	55.36%	778,597	56.58%
Point of service	23,776	13.82%	178,510	11.53%	45,378	13.19%	156,908	11.40%
Indemnity	13,218	7.68%	181,335	11.71%	31,063	9.03%	163,490	11.88%
Missing/unknown	1,659	0.96%	21,306	1.38%	3,558	1.03%	19,407	1.41%
Payer type								
Commercial	125,980	73.24%	1,156,587	74.71%	253,795	73.77%	1,028,772	74.76%
Medicaid	3,154	1.83%	17,382	1.12%	6,019	1.75%	14,517	1.05%
Medicare	18,213	10.59%	91,170	5.89%	31,044	9.02%	78,339	5.69%
Self	19,946	11.60%	213,595	13.80%	42,432	12.33%	191,109	13.89%
Medicare Gap	3,987	2.32%	60,944	3.94%	9,317	2.71%	55,614	4.04%
Missing/unknown	724	0.42%	8,359	0.54%	1,412	0.41%	7,671	0.56%
CCI Score^a^								
Mean (SD)	4.27 (3.04)		2.07 (1.68)		3.66 (2.75)		1.95 (1.55)	
CCI < 2	32,217	18.73%	879,610	56.82%	88,195	25.64%	823,632	59.86
CCI ≥ 2	139,787	81.27%	668,427	43.18%	255,824	74.36%	552,390	40.14
Charlson comorbidities								
Myocardial infarction	20,594	11.97%	32,411	2.09%	29,040	8.44%	23,965	1.74%
Congestive heart failure	36,022	20.94%	84,121	5.43%	55,756	16.21%	64,387	4.68%
Peripheral vascular disease	21,069	12.25%	68,687	4.44%	34,510	10.03%	55,246	4.01%
Cardiovascular disease	31,358	18.23%	109,220	7.06%	53,770	15.63%	86,808	6.31%
Dementia	2,419	1.41%	11,390	0.74%	4,765	1.39%	9,044	0.66%
Chronic pulmonary disease	49,776	28.94%	213,749	13.81%	89,955	26.15%	173,570	12.61%
Rheumatological disease	8,821	5.13%	30,566	1.97%	15,119	4.39%	24,268	1.76%
Peptic ulcer disease	5,174	3.01%	13,678	0.88%	8,643	2.51%	10,209	0.74%
Mild liver disease	3,654	2.12%	6,305	0.41%	5,380	1.56%	4,579	0.33%
Diabetes without chronic complications	169,829	98.74%	1,525,023	98.51%	339,677	98.74%	1,355,175	98.48%
Diabetes with chronic complications	45,140	26.24%	219,433	14.17%	83,992	24.41%	180,581	13.12%
Paraplegia	3,765	2.19%	4,538	0.29%	5,139	1.49%	3,164	0.23%
Renal impairment	17,789	10.34%	29,344	1.90%	26,180	7.61%	20,953	1.52%
Cancer	34,466	20.04%	101,118	6.53%	54,085	15.72%	81,499	5.92%
Severe liver disease	39,130	22.75%	113,367	7.32%	66,117	19.22%	86,380	6.28%
Metastatic cancer	9,830	5.71%	7,726	0.50%	12,346	3.59%	5,210	0.38%
HIV/AIDS	1,094	0.64%	2,050	0.13%	1,693	0.49%	1,451	0.11%
Antidiabetic agents received on Index								
Glucagon-like peptide-1	991	0.58%	7,500	0.48%	2,390	0.69%	6,101	0.44%
Dipeptidyl peptidase-4	629	0.37%	6,071	0.39%	1,291	0.38%	5,409	0.39%
Biguanides	21,285	12.37%	218,752	14.13%	46,060	13.39%	193,977	14.10%
Sulfonylureas	14,169	8.24%	122,570	7.92%	29,290	8.51%	107,449	7.81%
Thiazolidinedione	9,976	5.80%	85,809	5.54%	23,079	6.71%	72,706	5.28%
Meglitinides	1,045	0.61%	7,266	0.47%	2,491	0.72%	5,820	0.42%
Alpha-glucosidase inhibitors	187	0.11%	1,435	0.09%	460	0.13%	1,162	0.08%
Insulin	14,220	8.27%	70,542	4.56%	29,921	8.70%	54,841	3.99%
Other antidiabetic agents	8,689	5.05%	79,749	5.15%	19,045	5.54%	69,393	5.04%

CCI = Charlson Comorbidity Index; SD = standard deviation.

^a^CCI score calculated in the 1-year post-index date period.

Patients in the top 10% of the cost distribution were found to be similar to patients in the top 20% of the cost distribution in terms of mean age, sex distribution, geographic region, health plan type, and payer type. The mean (SD) CCI score was greater for patients in the top 10% of the cost distribution (4.3 [3.0]), with a greater percentage of these patients having nearly all of the individual CCI components, than for patients in the top 20% of the cost distribution (2.1 [1.7]). Additionally, approximately the same percentage of patients in the top 10% of the cost distribution received oral antidiabetic medications and insulin as in the top 20% of the cost distribution.

The strongest predictor of being an HC patient (either in the top 20% or top 10% of the cost distribution) was having a CCI score ≥ 2 (odds ratio [OR] for top 20% regression vs. top 10% regression: 3.9 vs. 4.9; both *P* < 0.0001) (Table 2). Additionally, a diagnosis of renal impairment (OR for top 20% regression vs. top 10% regression: 2.2 vs. 2.4; both *P* < 0.0001), a diagnosis of obesity (OR for top 20% regression vs. top 10% regression: 2.0 vs. 2.1; both *P* < 0.0001), receipt of insulin (OR for top 20% regression vs. top 10% regression: 2.7 vs. 2.1; both *P* < 0.0001), and a diagnosis of hypertension (OR for top 20% regression vs. top 10% regression: 1.5 vs. 1.6; both *P* < 0.0001) were all found to be associated with a significant increase in the odds of being an HC patient.

**Table 2:** **Predictors of being an HC T2DM patient, among all patients with T2DM**^**a**^

**Variable**	**Cohort**							
	**Top 10% of all-cause costs**				**Top 20% of all-cause costs**			
	**Odds ratio**	**Lower 95% CI**	**Upper 95% CI**	***P *** **value**	**Odds ratio**	**Lower 95% CI**	**Upper 95% CI**	***P *** **value**
Age, in years (vs. 55+ years)								
< 35	1.128	1.098	1.159	< 0.0001	1.311	1.286	1.337	< 0.0001
35-44	1.063	1.043	1.085	< 0.0001	1.094	1.078	1.110	< 0.0001
45-54	1.200	1.184	1.216	< 0.0001	1.179	1.167	1.191	< 0.0001
Female (vs. male)	1.023	1.012	1.034	< 0.0001	1.175	1.165	1.184	< 0.0001
Geographic region (vs. East)								
South	0.814	0.800	0.827	< 0.0001	0.763	0.754	0.773	< 0.0001
Midwest	1.166	1.150	1.183	< 0.0001	1.156	1.144	1.168	< 0.0001
West	1.107	1.088	1.128	< 0.0001	1.047	1.033	1.062	< 0.0001
Health plan type (vs. health maintenance organization)								
Preferred provider organization	1.091	1.074	1.108	< 0.0001	1.041	1.029	1.054	< 0.0001
Point of service	1.141	1.119	1.163	< 0.0001	1.080	1.063	1.096	< 0.0001
Indemnity	0.510	0.499	0.521	< 0.0001	0.608	0.598	0.618	< 0.0001
Payer type (vs. commercial)								
Medicaid	1.221	1.170	1.274	< 0.0001	1.198	1.157	1.239	< 0.0001
Medicare or Medicare Gap	1.070	1.050	1.089	< 0.0001	0.978	0.964	0.992	0.0024
Self	0.885	0.868	0.902	< 0.0001	0.967	0.953	0.981	< 0.0001
Charlson Comorbidity Index score ≥ 2 (vs. < 2)	4.896	4.832	4.961	< 0.0001	3.908	3.873	3.944	< 0.0001
Renal impairment diagnosis (vs. no renal impairment diagnosis)	2.368	2.333	2.404	< 0.0001	2.179	2.150	2.208	< 0.0001
Hypertension diagnosis (vs. no hypertension diagnosis)	1.625	1.602	1.648	< 0.0001	1.519	1.504	1.535	< 0.0001
Obesity diagnosis (vs. no obesity diagnosis)	2.106	2.076	2.13741	< 0.0001	2.033	2.009	2.056	< 0.0001
Antidiabetic treatment (vs. no treatment)								
Received insulin	2.098	2.068	2.128	< 0.0001	2.744	2.7142	2.775	< 0.0001
Received oral antidiabetic agents only	1.110	1.097	1.124	< 0.0001	1.283	1.271	1.294	< 0.0001

CI = confidence interval; HC = high cost; T2DM = type 2 diabetes mellitus.

^a^28,100 patients were excluded from the regression analysis because they were missing age, sex, health plan, or payer information.

Patients in the top 20% of the cost distribution had total all-cause costs that were, on average, $32,179 more than the costs accrued by patients in the bottom 80% of the cost distribution (mean [SD] all-cause costs, top 20% vs. bottom 80%: $35,596 [$50,903] vs. $3,417 [$2,775]) (Table 3). Furthermore, patients in the top 10% of the cost distribution had total all-cause costs that were, on average, $51,794 more than the costs accrued by patients in the bottom 90% of the cost distribution (i.e., mean [SD] all-cause costs, top 10% vs. bottom 90%: $56,468 [$65,604] vs. $4,674 [$4,504]. Additionally, all-cause costs for patients in the top 10% of the cost distribution were approximately $20,872 more than all-cause costs for patients in the top 20% of the cost distribution.

**Table 3:** Summary of overall health care utilization and costs during the 12-month follow-up period, by cohort

	**Cohort**							
	**Top 10% of costs**		**Bottom 90% of costs**		**Top 20% of costs**		**Bottom 80% of costs**	
Inpatient services								
Had ≥ 1 hospital admission (n, %)	127,553	74.16%	166,729	10.77%	192,070	55.83%	102,212	7.43%
Had ≥ 2 hospital admissions (n, %)	59,199	34.42%	31,289	2.02%	72,263	21.01%	18,225	1.32%
Mean number of admissions (SD)	1.44	(1.62)	0.14	(0.45)	0.97	(1.36)	0.09	(0.37)
Mean (SD) inpatient days^a^	13.81	(24.88)	7.07	(39.57)	11.65	(22.04)	6.89	(49.31)
Mean (SD) total costs	$24,766	($48,149)	$427	($1,739)	$13,618	($35,945)	$171	($805)
SNF stays								
Had ≥ 1 SNF admission (n, %)	10,410	6.05%	8,987	0.58%	14,047	4.08%	5,350	0.39%
Mean number of SNF admissions (SD)	0.10	(0.49)	0.01	(0.14)	0.07	(0.41)	0.01	(0.10)
Mean (SD) SNF days^a^	33.70	(44.70)	48.52	(203.18)	42.75	(135.81)	34.84	(157.98)
Mean (SD) total costs	$525	($3,532)	$20	($389)	$323	($2,612)	$7	($180)
ED visits								
Had ≥ 1 ED visit (n, %)	103,348	60.08%	355,475	22.96%	183,931	53.47%	274,892	19.98%
Mean number of ED visits (SD)	5.31	(14.32)	1.29	(4.60)	4.26	(11.65)	1.05	(3.90)
Mean (SD) total costs	$888	($2,283)	$136	($505)	$659	($1,791)	$99	($370)
Office visits								
Had ≥ 1 office visit (n, %)	168,403	97.91%	1,505,071	97.22%	336,935	97.94%	1,336,539	97.13%
Mean number of office visits (SD)	26.00	(20.97)	12.72	(12.11)	23.73	(19.25)	11.62	(10.86)
Mean (SD) total costs	$5,827	($13,092)	$1,155	($1,440)	$4,246	($9,562)	$967	($1,098)
Pharmacy								
Had ≥ 1 prescription (n, %)	150,474	87.48%	1,225,785	79.18%	302,929	88.06%	1,073,330	78.00%
Mean number of prescriptions (SD)	53.72	(44.11)	27.99	(28.83)	50.37	(41.21)	25.61	(26.56)
Mean (SD) total costs	$4,854	($12,486)	$1,428	($2,162)	$4,190	($9,270)	$1,166	($1,660)
Outpatient hospital visits								
Had ≥ 1 outpatient visit (n, %)	131,885	76.68%	844,910	54.58%	257,619	74.89%	719,176	52.26%
Mean number of outpatient visits (SD)	43.33	(82.58)	9.15	(20.37)	32.64	(65.27)	7.55	(16.13)
Mean (SD) total costs	$8,763	($22,823)	$767	($1,816)	$5,807	($16,625)	$507	($1,136)
Laboratory services								
Had ≥ 1 laboratory service (n, %)	92,529	53.79%	655,781	42.36%	178,840	51.99%	569,470	41.39%
Mean number of laboratory services (SD)	13.13	(30.85)	5.50	(11.32)	10.72	(24.64)	5.15	(10.57)
Mean (SD) total costs	$247	($810)	$75	($210)	$194	($630)	$66	($179)
OOP services								
Had ≥ 1 OOP services (n, %)	153,936	89.50%	834,883	53.93%	293,499	85.31%	695,320	50.53%
Mean number of OOP services (SD)	46.30	(95.37)	8.49	(22.41)	34.05	(75.59)	6.82	(16.91)
Mean (SD) total costs	$10,598	($29,858)	$666	($1,633)	$6,559	($21,633)	$434	($1,016)
Total health care utilization								
Had ≥ 1 medical encounter (n, %)	172,004	100.00%	1,548,037	100.00%	344,019	100.00%	1,376,022	100.00%
Mean number of encounters (SD)	189.33	(150.55)	65.29	(51.82)	156.81	(123.32)	57.92	(42.74)
Mean (SD) total costs	$56,468	($65,604)	$4,674	($4,504)	$35,596	($50,903)	$3,417	($2,775)

ED = emergency department; OOP = other outpatient; SD = standard deviation; SNF = skilled nursing facility.

^a^Mean inpatient and SNF days estimated among only those patients with at least 1 unique admission.

All-cause inpatient visits were responsible for approximately 42% of the difference in all-cause costs between patients in the top 20% and patients in the bottom 80% of the cost distribution, with 55.8% of patients in the top 20% of the cost distribution having at least one all-cause hospitalization and 21.0% of patients having two or more hospitalizations compared with 7.4% of patients in the bottom 80% of the cost distribution having at least one all-cause hospitalization and 1.3% of patients having two or more hospitalizations. Similarly, all-cause inpatient visits were responsible for approximately 47% of the difference in costs between patients in the top 10% and patients in the bottom 90% of the distribution, with 74.2% of patients in the top 10% of the distribution having at least one all-cause hospitalization and 34.4% of patients having two or more hospitalizations, compared with only 10.8% of patients in the bottom 90% of the distribution having at least one all-cause hospitalization and 2.0% of patients having two or more hospitalizations.

All-cause outpatient hospital visits contributed to approximately 16.5% of the difference in costs between patients in the top 20% and patients in the bottom 80% of the cost distribution, with 74.9% of patients in the top 20% of the cost distribution having at least one all-cause outpatient hospital visit compared with 52.3% of patients in the bottom 80% of the cost distribution. Outpatient hospital visits had a similar contribution to the difference in costs between patients in the top 10% and patients in the bottom 90% of the cost distribution. Specifically, all-cause outpatient hospital visits contributed to approximately 15.4% of the difference in all-cause costs between patients in the top 10% of the cost distribution and patients in the bottom 90% of the cost distribution, with 76.7% of patients in the top 10% of the cost distribution having at least one all-cause outpatient hospital visit, compared with 54.6% of patients in the bottom 90% of the all-cause cost distribution.

All-cause prescription fills and all-cause office visits contributed to 9.4% and 10.2% of the difference in all-cause costs among patients in the top 20% and bottom 80%, respectively, of the cost distribution. All-cause prescription fills and all-cause office visits contributed to 6.6% and 9.0% of the difference among patients in the top 10% and bottom 90%, respectively, of the cost distribution. However, patients in both the top 20% and top 10% of the cost distribution filled approximately twice as many prescriptions and had almost double the number of physician office visits, compared with patients in the bottom 80% and bottom 90% of the cost distribution (top 20% vs. bottom 80%: 50.4 vs. 25.6 prescription fills; 23.7 vs. 11.6 office visits: top 10% vs. bottom 90%: 53.7 vs. 28.0 prescription fills; 26.0 vs. 12.7 office visits).

**Figure 1:**
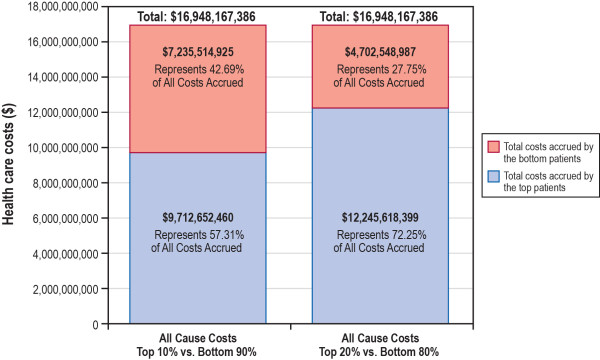
Descriptive summary of all-cause health care costs during the 12-month follow-up period.

**Figure 2:**
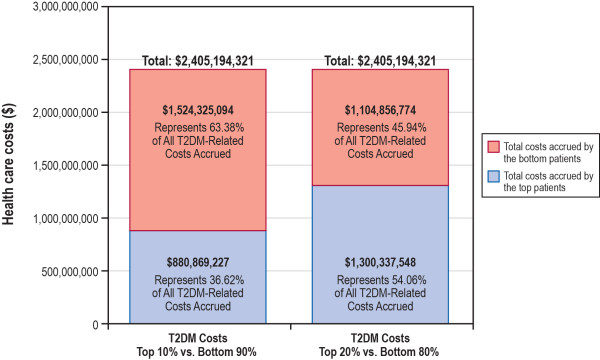
**Descriptive summary of T2DM-related health care costs during the 12-month follow-up period.** T2DM = type 2 diabetes mellitus.

Health care costs related to T2DM were, on average, $2,977 more for patients in the top 20% of the cost distribution than for patients in the bottom 80% of the cost distribution and $4,136 more for patients in the top 10% of the cost distribution than for patients in the bottom 90% of the cost distribution (Table 4). Mean (SD) T2DM-related costs among patients in the top 20% of the cost distribution were $3,780 ($8,530), which represented 10.6% of all-cause costs; T2DM-related costs among patients in the bottom 80% of the cost distribution were $803 ($1,065), which represented 23.5% of all-cause costs. Similarly, mean (SD) T2DM-related costs among patients in the top 10% of the cost distribution were $5,121 ($11,575), which represented 9.1% of all-cause costs; T2DM-related costs among patients in the bottom 90% of the cost distribution were $985 ($1,469), which represented 21.1% of all-cause costs. Unlike all-cause costs, the biggest difference in T2DM-related costs between patients in the top 20% and 10% and patients in bottom 80% and 90% of the cost distribution was outpatient hospital visits, which accounted for approximately 25% of the cost difference in both groups.The entire T2DM population included in this study (N = 1,720,041) accrued all-cause costs of approximately $17 billion (Figures 1 and 2). The top 10% of patients accrued costs of more than $9.7 billion, which represented more than 57% of the costs accrued by this population. The top 20% of patients accrued costs of more than $12 billion, which represented more than 72% of the costs accrued by this population. In the overall population of patients, over $2.4 billion of the total all-cause costs could be directly linked to T2DM (i.e., 14.2% of all-cause costs accrued by this population were attributable directly to T2DM). The top 10% of patients accrued T2DM-related costs of $880 million, which represented 36.6% of the total T2DM-related costs, while the top 20% of patients accrued T2DM-related costs of $1.3 billion, which represented 54.1% of the total T2DM-related costs.

**Table 4:** Summary of diabetes-related health care utilization and costs during the 12-month follow-up period, by cohort

	**Cohort**							
	**Top 10% of costs**		**Bottom 90% of costs**		**Top 20% of costs**		**Bottom 80% of costs**	
Diabetes-related inpatient stays								
Had ≥ 1 hospital admission (n, %)	30,310	17.62%	27,729	1.79%	41,220	11.98%	16,819	1.22%
Mean number of hospital admissions (SD)	0.23	(0.60)	0.02	(0.15)	0.15	(0.48)	0.01	(0.12)
Mean (SD) inpatient days^a^	6.93	(10.48)	4.29	(7.60)	6.28	(9.68)	4.17	(8.16)
Mean costs (SD)	$677	($5,515)	$20	($331)	$387	($3,953)	$10	($194)
Diabetes-related SNF stays (n, %)								
Had ≥ 1 SNF admission	1,802	1.05%	1,405	0.09%	2,468	0.72%	739	0.05%
Mean number of SNF admissions (SD)	0.01	(0.14)	0.00	(0.04)	0.01	(0.12)	0.00	(0.03)
Mean (SD) SNF days^a^	12.96	(31.56)	22.29	(139.07)	17.53	(105.76)	15.41	(43.85)
Mean costs (SD)	$14	($424)	$1	($76)	$9	($332)	$0	($37)
Diabetes-related ED visits								
Had ≥ 1 ED visit (n, %)	39,813	23.15%	132,909	8.59%	70,699	20.55%	102,023	7.41%
Mean number of ED visits (SD)	1.36	(5.88)	0.38	(2.37)	1.14	(5.03)	0.31	(2.07)
Mean costs (SD)	$232	($1,003)	$42	($277)	$182	($825)	$31	($204)
Diabetes-related office visits								
Had ≥ 1 office visit (n, %)	137,104	79.71%	1,273,378	82.26%	277,094	80.55%	1,133,388	82.37%
Mean number of office visits (SD)	4.28	(4.59)	3.42	(3.32)	4.23	(4.39)	3.33	(3.18)
Mean costs (SD)	$469	($1,290)	$245	($357)	$424	($1,002)	$228	($314)
Diabetes-related pharmacy								
Had ≥ 1 prescription (n, %)	107,729	62.63%	818,451	52.87%	219,248	63.73%	706,932	51.38%
Mean number of prescriptions (SD)	6.98	(8.57)	5.49	(7.72)	7.29	(8.78)	5.22	(7.51)
Mean costs (SD)	$636	($1,660)	$356	($806)	$702	($1,511)	$304	($694)
Diabetes-related outpatient hospital visits								
Had ≥ 1outpatient visit (n, %)	69,070	40.16%	411,072	26.55%	131,277	38.16%	348,865	25.35%
Mean number of outpatient visits (SD)	6.18	(18.89)	2.46	(7.63)	5.23	(16.47)	2.23	(6.49)
Mean (SD) total costs	$1,204	($5,433)	$145	($652)	$849	($4,007)	$101	($408)
Diabetes-related laboratory services								
Had ≥ 1 laboratory service (n, %)	51,096	29.71%	439,858	28.41%	101,715	29.57%	389,239	28.29%
Mean number of laboratory services (SD)	3.52	(9.92)	2.76	(6.48)	3.35	(8.82)	2.71	(6.32)
Mean (SD) total costs	$48	($204)	$29	($90)	$43	($169)	$28	($85)
Diabetes-related OOP services								
Had ≥ 1 OOP service (n, %)	80,576	46.85%	414,844	26.80%	147,877	42.99%	347,543	25.26%
Mean number of OOP services (SD)	8.11	(24.22)	2.29	(7.32)	6.33	(19.66)	2.01	(6.09)
Mean costs (SD)	$1,842	($7,754)	$147	($659)	$1,182	($5,634)	$100	($398)
Diabetes-related total health care utilization								
Had ≥ 1 medical encounter (n, %)	168,326	97.86%	1,541,265	99.56%	338,383	98.36%	1,371,208	99.65%
Mean number of encounters (SD)	30.68	(37.16)	16.83	(16.26)	27.72	(31.67)	15.84	(14.58)
Mean costs (SD)	$5,121	($11,575)	$985	($1,469)	$3,780	($8,530)	$803	($1,065)

ED = emergency department; OOP = other outpatient; SD = standard deviation; SNF = skilled nursing facility.

^a^Mean inpatient and SNF days estimated among only patients with at least 1 unique admission.

## Discussion

This study examined patients with T2DM in a large, managed care population and quantified differences in health care costs by categories of cost distributions. Patients were identified as being HC if their total care costs fell in the top 10% or the top 20% of the total cost distribution. Patients in the top 10% of the total cost distribution accrued annual per-patient health care costs that were on average $50,000 more than the annual per-patient health care costs accrued by patients in the bottom 90% of the total cost distribution. Similarly, patients in the top 20% of the total cost distribution accrued annual per-patient health care costs that were over $32,000 more than the annual per-patient health care costs accrued by patients in the bottom 80% of the total cost distribution. Multivariable logistic regression models found that patients were significantly more likely to be in the top 10% or the top 20% of the total cost distribution if they had a CCI score ≥ 2; had received a diagnosis of renal impairment, obesity, or hypertension; or were treated with insulin.

Data drawn from the Medical Expenditure Panel Survey have previously shown that a small percentage of patients typically account for a large percentage of health care costs. Specifically, using data from 1999, the survey found that, in the general community population, more than half of the total health care spending was accrued by only 5% of the population [16]. The Olin study supports the rule of thumb that 20% of patients consume 80% of health care resources. Additionally, Conwell and Cohen, using data from a 2002 US noninstitutionalized population, found that exactly 20% of patients accrued 80% of costs [14]. Similarly, we found that patients in the top 20% of the total cost distribution accrued costs of $12.2 billion annually, which represented 72% of the total costs accrued by the T2DM population. Additionally, we found that patients in the top 10% of the total cost distribution accrued costs of $9.7 billion annually, which represented 57% of the total costs accrued by the T2DM population.

This study used methodology similar to the approach described by Etemad and McCollam in an article examining predictors of HC managed care patients with acute coronary syndrome [13]. Etemad and McCollam identified patients with newly onset acute coronary syndrome and assessed these patients’ health care costs over 12 months after disease onset. The authors classified patients as being HC if the patients accrued costs in the top 20% of the population; multivariable regression analyses were estimated to assess predictors of being an HC patient. Similar to our study, many of the factors associated with being an HC patient in the Etemad analysis were nonmodifiable comorbidities such as hypertension, diabetes, or pulmonary disease.

Etemad and McCollam also observed that an initial hospitalization for acute coronary syndrome had costs that were equal to nearly two-thirds of the costs accrued in the entire year following hospital discharge. Although our study used a slightly different methodology (we examined all inpatient stays vs. a single initial inpatient stay) to that of Etemad and McCollam, we found that approximately 40% of the health care costs accrued in HC patients were associated with inpatient visits.

Hartmann [17] examined patients in the top decile of health care spending, using German health insurance information. Consistent with our analysis, Hartmann found that the highest health care expenses for patients were incurred in the inpatient sector, with over 80% of all HC patients having at least one hospital admission (compared with 74.2% in our analysis) [17]. Additionally, Hartmann [17] found that the reasons for the hospitalization differed based on patient age and sex, which further highlights the facts that HC patients require care tailored to their unique situation and that no single intervention exists that will reduce health care costs among all patients.

A previous study examining Medicare patients with T2DM found that interventions aimed at diabetes have not differed based on comorbid illness burden [18]. Our analysis found that patients with a higher comorbidity burden and more concomitant conditions were significantly more likely to be HC. Therefore, from the perspective of a payer, one practical implication of the present analysis is that it may make sense to provide those patients who have the most comorbidities and concomitant conditions (i.e., those patients who are at the greatest risk of being HC) with additional patient care tailored at treating the comorbidity or concomitant condition (e.g., weight loss programs for obese patients).

Sensitivity analyses were conducted, examining the 926,180 patients who received an antidiabetic medication (i.e., either an oral antidiabetic or insulin). In this subpopulation of treated T2DM patients, those with costs greater than $22,646 comprised the top 10th percentile (vs. $20,528 in the overall T2DM population), while patients with costs greater than $12,349 comprised the top 20th percentile (vs. $10,901 in the overall T2DM population). We found that there were no differences in patient demographics between the overall study sample and those patients who received antidiabetic medication. Predictors of being an HC T2DM patient were the same for the treated and overall T2DM populations. Specifically, in the treated T2DM population, having a CCI score greater than or equal to 2 was the strongest predictor of being an HC patient (OR = 4.862; *P* < 0.001), followed by a renal impairment diagnosis (OR = 2.369; *P* < 0.001), an obesity diagnosis (OR = 1.991; *P* < 0.001), or receipt of insulin (OR = 1.897; *P* < 0.001). Treated patients in the top 10% of the cost distribution accrued approximately $53,917 more in health care costs versus treated patients in the bottom 90% of the cost distribution (vs. $51,794 more in costs in the overall T2DM population), with the largest difference in costs attributable to inpatient stays. Additionally, treated patients in the top 10% of the cost distribution accrued costs of over $5.5 billion, which represented 54.1% of all costs accrued by the treated T2DM population (vs. 57.3% among all T2DM patients). Treated patients in the top 20% of the cost distribution accrued costs of over $7.0 billion, which represented 69.0% of all costs accrued by the treated T2DM population (vs. 72.3% among all T2DM patients).

This study has several limitations common to most retrospective database studies. First, it was not possible to confirm diagnoses for T2DM, renal impairment, hypertension, or obesity. No laboratory data were available to further assess the level of renal impairment, and no information was available in the database regarding patients’ height or weight. Thus, rates of obesity and renal impairment reported in the analysis are likely underestimated. Additionally, no information was available regarding blood glucose or glycated hemoglobin values, so the effect of glucose control on costs could not be assessed. Logistic regression model specifications were limited to the data available, and additional predictors of being an HC patient may exist (e.g., increased glycated hemoglobin value). Because this study used retrospective administrative claims, it was not feasible to assess the effect of an intervention (e.g., change in diabetes medication) on costs. Further, because our study used data from a managed care population, results may not be applicable to Medicaid, Medicare, or uninsured patients.

The goal of this study was to provide payers with a means of identifying patients who are at increased risk for becoming HC, using real-world data. Once these patients are identified, personalized interventions could be developed that may decrease the likelihood of the patient becoming HC. Interventions might include extra office visits for comorbid conditions, structured weight loss programs, or increased pharmacotherapy for glucose control. Economic evaluations to examine the cost-benefit structure of developing such interventions would be informative.

## Conclusions

This study examined health care resource utilization and costs in a large, real-world, managed care population. In conclusion, it was found that patients with T2DM who make up the top 10% of a cost distribution for T2DM accrue, on average, 12 times more total annual health care costs than patients who make up the bottom 90% of the cost distribution. Further, T2DM patients who make up the top 20% of the cost distribution accrue, on average, 11 times more health care costs than patients who make up the bottom 80% of the cost distribution. Obesity and progression to insulin were found to predict the odds of being an HC patient and are two modifiable factors for T2DM patients. Further research is needed to explore potential interventions to reduce the likelihood that a patient becomes HC. Our study also found that cost of a hospitalization was the largest component of HC patients’ total care costs. Reducing all-cause hospitalizations in patients with T2DM through interventions aimed at better management of T2DM (e.g., outpatient management, lifestyle changes) may help to reduce costs.

## Abbreviations

CCI: Charlson Comorbidity Index; HC: High cost; ICD-9-CM: International Classification of Diseases, Ninth Revision, Clinical Modification; NHC: Not high cost; OR: Odds ratio; SD: Standard deviation; T2DM: Type 2 diabetes mellitus; US: United States; CI: Confidence interval; ED: Emergency department; HMO: Health maintenance organization; OOP: Other outpatient; PPO: Preferred provider organization; SNF: Skilled nursing facility.

## Competing interests

The authors declare that they have no competing interests.

## Authors’ contributions

JLM participated in the design of the study, carried out the research, performed the data analysis, and drafted the manuscript. SP conceived the study design, carried out the research, reviewed study results, and provided senior review of the manuscript. KB participated in the design of the study, carried out the research, reviewed study results, and provided senior review of the manuscript. JG participated in the design of the study, reviewed study results, and provided senior review of the manuscript. SDC participated in the design of the study, assisted with the data analysis, and helped to draft the manuscript. All authors read and approved the final manuscript.

## Authors’ information

JLM and SDC are employees of RTI Health Solutions, a research organization hired by AstraZeneca to conduct the analysis described in this manuscript. Neither JLM nor SDC have any competing interests to declare. SP is an employee of AstraZeneca. KB and JG are employees of Bristol-Myers Squibb.
